# Delayed diagnosis of *Plasmodium vivax* malaria and associated factors among Thai patients and patients from neighboring countries at the Hospital for Tropical Diseases, Bangkok, Thailand

**DOI:** 10.1007/s15010-026-02930-8

**Published:** 2026-08-17

**Authors:** Sakarn Charoensakulchai, Noppadon Tangpukdee, Polrat Wilairatana, Srivicha Krudsood, Wiwat Chancharoenthana, Sant Muangnoicharoen, Panita Looareesuwan

**Affiliations:** 1 Thai Travel Clinic, Hospital for Tropical Diseases, Faculty of Tropical Medicine, Mahidol University, Bangkok, 10400 Thailand; 2 Department of Preventive and Social Medicine, Faculty of Medicine, Srinakharinwirot University, Nakhon Nayok, 26120 Thailand; 3 Department of Clinical Tropical Medicine, Faculty of Tropical Medicine, Mahidol University, Bangkok, 10400 Thailand; 4 Clinical Malaria Research Unit, Faculty of Tropical Medicine, Mahidol University, Bangkok, 10400 Thailand; 5 Department of Tropical Hygiene, Faculty of Tropical Medicine, Mahidol University, Bangkok, 10400 Thailand; 6 Department of Social and Environmental Medicine, Faculty of Tropical Medicine, Mahidol University, Bangkok, 10400 Thailand

**Keywords:** Delayed care seeking, Delayed diagnosis, Urban, Imported malaria, Misdiagnosis, *P. vivax*

## Abstract

**Purpose:**

*Plasmodium vivax* is the predominant malaria species in Southeast Asia, yet diagnosis may be delayed in non-endemic urban settings where clinicians encounter malaria infrequently. This study described patient characteristics and identified factors associated with delayed diagnosis of *P. vivax* malaria at the Hospital for Tropical Diseases (HTD), Bangkok, Thailand.

**Methods:**

A retrospective study was conducted among patients admitted with *P. vivax* malaria at HTD between 2010 and 2024. Data collected included demographics, travel history, illness and healthcare-seeking history prior to admission, clinical manifestations, laboratory findings, and outcomes. Characteristics of patients with and without delayed diagnosis were compared. Logistic regression was used to identify factors independently associated with delayed diagnosis.

**Results:**

Among 384 patients, 58.3% experienced delayed diagnosis (> 4 days from symptom onset). Care-seeking delay (> 3 days) occurred in 73.7%, medical diagnostic delay (> 1 day) in 14.1%, and misdiagnosis at the first healthcare facility in 25.5%. Independent factors of delayed diagnosis included age 45–54 years (adjusted odds ratio [AOR] 2.79; 95% CI 1.12–6.94; *p* = 0.027); working as an employee/office worker/business owner (AOR 4.93; 95% CI 1.19–20.50; *p* = 0.028); no prior history of malaria (AOR 2.36; 95% CI 1.26–4.42; *p* = 0.008); and misdiagnosis at the first healthcare facility (AOR 2.99; 95% CI 1.52–5.89; *p* = 0.002).

**Conclusion:**

Delayed *P. vivax* malaria diagnosis was common in this urban near-elimination setting, with care-seeking delay contributing more than medical diagnostic delay. Age 45–54 years, office-based occupation, no prior history of malaria, and initial misdiagnosis were independently associated with delayed diagnosis. Malaria should be considered in febrile patients with relevant epidemiological exposure.

**Supplementary Information:**

The online version contains supplementary material available at https://doi.org/10.1007/s15010-026-02930-8.

## Introduction

Malaria remains a major global public health burden, with an estimated 280 million cases in 2025 [1]. *Plasmodium vivax* is one of the leading causes of malaria worldwide, with one-third of the world’s population at risk of infection [2]. The highest burden of *P. vivax* infection is in South Asia, Southeast Asia, and South America [1, 3]. 

*P. vivax* elimination is particularly more challenging than *P. falciparum* due to its distinctive biological and epidemiological features, including dormant liver stages (hypnozoites), early appearance of transmissible sexual stages, high genetic diversity, and low-density infections which can sustain transmission and hinder diagnosis [4–6]. Furthermore, rising human mobility has contributed to a rise in imported cases in areas approaching elimination [7, 8]. In countries where both *P. vivax* and *P. falciparum* coexist, the proportion of malaria cases caused by *P. vivax* has been steadily rising [9]. Moreover, among imported malaria cases in several countries, a large proportion of cases were due to *P. vivax* [10–12]. 

In Thailand, malaria incidence has declined substantially, yet *P. vivax* now accounts for over 90% of cases in recent years, with most infections concentrated along the Myanmar and Cambodia borders. A large proportion of cases were imported from neighboring countries with imported *P. vivax* case showed a steadily increase from 586 cases in 2021 to 7,246 in 2023 and 4,516 in 2025 [13]. Some of these cases were imported to inner, urbanized areas in Thailand [10, 13]. In urban areas, particularly Bangkok, most *P. vivax* cases were imported, either internationally from neighboring countries or domestically from provinces bordering Myanmar and Cambodia [13]. Similar epidemiological shifts have been observed in other near-elimination settings, where reduced clinician familiarity with malaria may contribute to misdiagnosis and delayed treatment [12, 14, 15]. Previous studies conducted in Spain and China showed that 46% and 57% of imported cases, respectively, were initially misdiagnosed at the first healthcare facility [16, 17]. 

Misdiagnosis and delayed diagnosis have been associated with double to triple the odds of severe malaria [16]. Patients who are initially misdiagnosed also experience a longer time from symptom onset to correct diagnosis, with more than 30% subsequently developing severe malaria [18]. Although these findings have been reported primarily in *P. falciparum* [16, 18], evidence suggests that delayed care-seeking and diagnosis are likewise associated with increased complications, such as respiratory distress, in *P. vivax* malaria [19]. 

Despite these trends, evidence on the frequency, patterns and determinants of delayed *P. vivax* diagnosis in low-transmission and urbanized areas remain limited. In particular, little is known about the relative contributions of patient-related care-seeking delay and healthcare-related medical diagnostic delay in settings approaching malaria elimination. This study aimed to characterize diagnostic delay among patients with *P. vivax* malaria in Bangkok, Thailand, quantify the contributions of care-seeking and diagnostic delays, and identify factors associated with delayed diagnosis.

## Methods

### Study design and site

This retrospective medical record review was conducted at the Hospital for Tropical Diseases (HTD), Faculty of Tropical Medicine, Mahidol University. The Hospital for Tropical Diseases is a national tertiary referral center for malaria and other tropical infectious diseases located in central Bangkok, Thailand, with approximately 250 inpatient beds. Hospital admission was determined by the treating physician based on clinical judgment.

### Study population

This study was a secondary analysis of an existing retrospective dataset of hospitalized patients with *P. vivax* malaria. The original dataset was developed for a separate research objective using medical records of patients admitted between 1st January 2010 and 31st December 2024. Patient records were archived as hard-copy records at the Clinical Malaria Research Unit, Faculty of Tropical Medicine, Mahidol University.

For the original dataset, patients aged ≥ 15 years with microscopically confirmed *P. vivax* mono-infection were eligible for inclusion. Patients with mixed malaria infection, systemic co-infections, or missing malaria count charts on the day of admission were excluded. Eligible patients were selected using stratified random sampling by year of admission. Demographic, clinical, laboratory, and treatment data were retrospectively extracted from the archived medical records using a standardized data collection form.

Among 604 eligible patients identified from hospital medical records, 400 participants were selected for the original dataset after applying the predefined eligibility criteria and stratified random sampling by year of admission.

For the present study, the original dataset was further restricted to Thai, Burmese, Cambodian, and Laotian patients. Patients of other nationalities were excluded because the objective of the present study was to compare delayed diagnosis between Thai patients and patients from neighboring countries. Restricting the analysis to these populations also minimized heterogeneity arising from patients from other regions who may differ in demographic and social characteristics that could influence diagnostic delay.

### Sample size calculation

The sample size was calculated using the prevalence of delayed malaria diagnosis reported by Zhang and colleagues (26.6%) [20]. Assuming a 95% confidence level and a 5% margin of error, the minimum required sample size was 301 patients. To account for potential missing data due to inherent retrospective design, this estimate was increased by 20%, yielding a target sample size of at least 362 patients.

### Data collection procedure

Eligible cases were selected using stratified random sampling by year of admission to ensure representation across the study period. Data were extracted and recorded in Microsoft Excel (Microsoft, Redmond, WA).

Collected variables included sex, age, nationality, occupation, comorbidities, previous malaria history, country of *P. vivax* malaria acquisition, and the purpose of travel to or residence in malaria-endemic areas. Information on illness and healthcare-seeking history prior to admission included the interval from symptom onset to the first healthcare contact (care-seeking delay), type of first healthcare facility visited, interval from first healthcare contact to confirmed *P. vivax* diagnosis (medical diagnostic delay), and interval from *P. vivax* diagnosis to administration of antimalarial treatment (treatment delay).

Clinical data recorded at presentation included body temperature, respiratory rate, blood pressure, pulse rate, and presenting symptoms. Laboratory variables included peripheral parasite count, presence of schizonts, complete blood count, liver and renal function tests, serum electrolytes, and hemoglobinuria. Data on malaria severity at admission and in-hospital clinical outcomes such as intensive care unit admission, and requirements for oxygen therapy or inotropic supports, were also collected.

### Operational definitions

Patients were classified as Thai if Thai nationality was documented in the medical records. Non-Thai patients were defined as patients of Myanmar, Lao, or Cambodian nationality, as recorded in the medical records.

The World Health Organization (WHO) defines care-seeking delay for *P. falciparum* malaria as presentation more than 24 h after symptom onset [21]. Consistent with previous studies of imported malaria, care-seeking delay was defined as an interval of more than 3 days between symptom onset and the first healthcare facility visit [20]. Medical diagnostic delay was defined as more than 24 h between the first healthcare visit and correct diagnosis of *P. vivax* malaria, and treatment delay as more than 24 h between diagnosis and initiation of antimalarial treatment [20, 21]. Total diagnostic delay was defined as the sum of care-seeking delay and medical diagnostic delay [20]. 

Because no universally accepted threshold exists for total delayed diagnosis of *P. vivax* malaria, two thresholds were evaluated. The primary outcome was total diagnostic delay, prespecified as > 4 days from symptom onset to confirmed diagnosis, consistent with the methodology of Zhang et al. [20] This threshold incorporated care-seeking delay (> 3 days from symptom onset to first healthcare visit) and medical diagnostic delay (> 1 day from first healthcare visit to confirmed diagnosis). As a sensitivity analysis, an alternative threshold for total diagnostic delay was determined post hoc using receiver operating characteristic (ROC) curve analysis, with severe malaria as the outcome. The ROC-derived optimal cut-off was > 6 days (Supplementary Materials S1 and S2). The 4-day threshold (> 4 days) was used as the primary definition and analysis given that it was defined a priori from an external source, thereby avoiding the risk of overfitting inherent to outcome-derived thresholds in retrospective designs, and because a more conservative threshold is operationally preferable for early identification of diagnostically delayed patients before disease progression, whereas ROC-derived 6-day threshold was evaluated in the secondary analysis.

Healthcare facilities were categorized as public hospitals, private hospitals, and primary care outpatient clinics. Public hospitals are government-owned hospitals which provides both inpatient and outpatient services. Private hospitals are privately owned hospitals providing inpatient and outpatient services. Primary care outpatient clinics are stand-alone healthcare facilities that provide first-contact ambulatory medical care without inpatient services and are not affiliated with hospitals.

Initial misdiagnosis was defined as any diagnosis other than *P. vivax* malaria at the first healthcare facility visited. Non-specific provisional or syndromic diagnoses documented in referral notes or medical records, including “fever,” “acute febrile illness,” and “fever of unknown origin,” as well as other initial diagnoses that were not *P. vivax* malaria, were classified as *P. vivax* malaria misdiagnoses. Cases in which malaria was diagnosed but the species was incorrectly identified were also classified as misdiagnosis. “Suspected *P. vivax* malaria” at first presentation was considered correct identification. All patients presenting to HTD underwent repeat peripheral blood smear examination for confirmation of malaria infection and species identification, irrespective of initial diagnosis.

### Statistical analysis

Statistical analyses were performed using SPSS version 29.0 (IBM Corporation, Armonk, NY). Categorical variables were summarized as frequencies and percentages. Differences between patients with and without total diagnostic delay were assessed using the Chi-square or Fisher exact test, as appropriate. Statistical significance was defined as a *p-*value of less than 0.05.

Because delay intervals were non-normally distributed, care-seeking delay, medical diagnostic delay, and treatment delay were summarized as medians and interquartile ranges (IQR) and compared between Thai and non-Thai patients using the Mann-Whitney *U*-test. The proportions of patients experiencing each type of delay were compared between Thai and non-Thai patients using Pearson’s Chi-square test.

Factors associated with total diagnostic delay were evaluated using logistic regression. Before multivariable modelling, multicollinearity among candidate variables was assessed using the variance inflation factors (VIFs). Variables with *p* < 0.20 in univariable analysis, together with variables identified a priori from previous literature, were entered in multivariable analysis. Adjusted odds ratios (AOR) and 95% confidence intervals (CI) were reported. Variables with *p* < 0.05 were considered independently associated with delayed diagnosis.

To preserve temporal relationship between predictors and the outcome, and because the objective was to identify factors associated with delayed diagnosis rather than to develop a prediction model, only demographic characteristics and information available before or at the initial clinical assessment, including the initial diagnosis, presenting symptoms, and vital signs, were considered for the regression analysis. Laboratory parameters obtained after presentation to the HTD were excluded because they were not routinely available at the time of the initial diagnostic decision and generally became available only after malaria had been confirmed by peripheral blood smear examination. Likewise, disease severity and post-admission clinical outcomes were not included because they represent potential consequences of delayed diagnosis rather than antecedent predictors. Their inclusion could therefore introduce temporal ambiguity and overadjustment.

To assess the robustness of the primary findings from a 4-day total diagnostic delay cut-off, a secondary logistic regression analysis was performed using an alternative definition of total diagnostic delay (> 6 days).

### Ethics considerations

As this retrospective study involved the review of existing medical records, the requirement for informed consent was waived by the Institutional Review Board of the Faculty of Tropical Medicine, Mahidol University (MUTM 2026-032-01). Permission to access medical records was obtained from the Hospital Director before data collection. To protect patient privacy and confidentiality, all data were de-identified before analysis. Personal identifiers were not included in the study database. Each participant was assigned a unique study identification code, and the study database was password-protected with access restricted to the principal investigator.

## Results

**Fig. 1:**
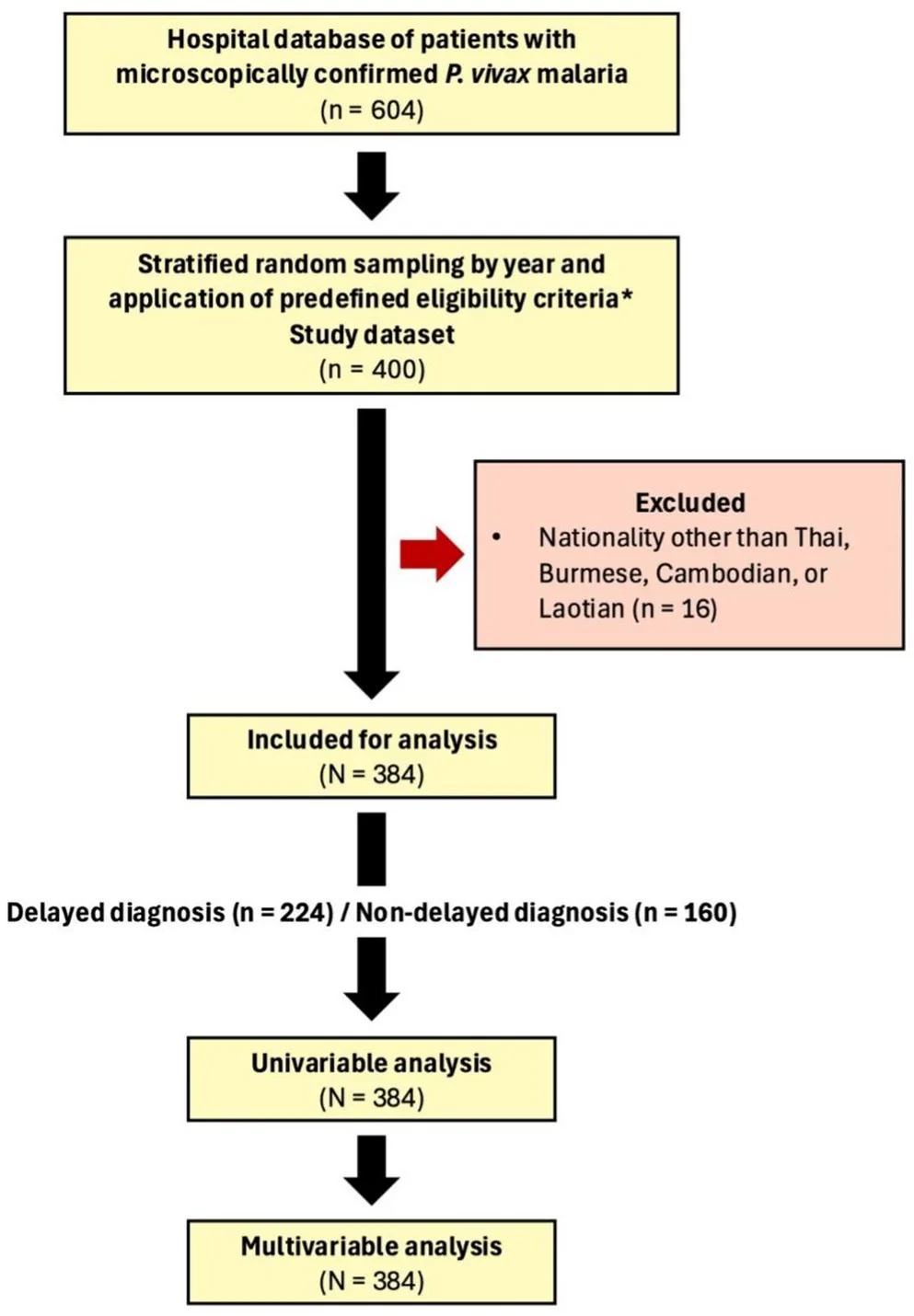
Study flow diagram. ^*^Footnote: Predefined eligibility criteria for the original dataset included patients aged ≥ 15 years with microscopically confirmed *P. vivax* malaria. Patients with mixed malaria infection, concurrent infection, or incomplete admission data were excluded

During the study period, 604 cases of *P. vivax* malaria were admitted. Of these, 400 cases were selected for the original dataset using stratified random sampling with pre-defined eligibility and exclusion criteria as mentioned earlier. Furthermore, in this study, we excluded 16 patients of nationalities other than Thai, Burmese, Laotian, or Cambodian. The final analysis included 384 patients. The study flow is presented in Fig. 1.

### Characteristics of patients

Most patients were male (84.9%), and aged 15–24 years (29.6%). Non-Thai patients accounted for 64.6% of the cohort, 67.2% worked as laborers and 91.1% had no comorbidities. A history of prior malaria infection was absent in 38.0% of patients. More than half of infections were acquired in Thailand (58.9%). Most patients (63.5%) were residents of malaria-endemic areas who travelled to Bangkok. The most common presenting symptoms were chills (87.0%), myalgia (56.8%), and headache (54.2%).

Total diagnostic delay was significantly more frequent among older patients (*p* = 0.047), Thai patients (*p* = 0.036), those employed as employees/office workers/business owners, and those working in forest-related occupations (including forest workers, patrol military and police personnel, and pilgrimage monks) (*p* = 0.009 for occupation overall). Delay was also more common among patients with no prior malaria infection (*p* < 0.001), and among those traveling to visit friends and relatives, tourism, or work-related purposes compared with non-delayed cases (*p* = 0.002). Baseline characteristics and group comparison between delayed and non-delayed diagnosis groups are presented in Table 1.

### Characteristics of care-seeking, diagnostic, and treatment delays in Thai and non-Thai patients

The overall median duration from symptom onset to the first medical visit was 4.0 days (IQR 3.0–6.0). There was no evidence of a difference between Thai and non-Thai patients (4.0 days [IQR 3.0–6.0] versus 4.0 days [IQR 3.0-6.8], respectively; Mann-Whitney U test *p* = 0.393). Overall, 73.7% experienced care-seeking delay, with comparable frequencies among Thai (73.5%) and non-Thai (73.8%) patients (*p* = 0.956).

The overall median duration from the first medical visit to the correct diagnosis of *P. vivax* malaria was 0.0 days (IQR 0.0–0.0) and was similar between Thai and non-Thai patients (Table 2). Overall, 14.1% of patients experienced medical diagnostic delay, which was more frequent among Thai patients (*p* < 0.001).

The median total diagnostic delay was 5.0 days (IQR 4.0–7.0), with no evidence of a difference in its distribution between the groups (Mann-Whitney U test *p* = 0.164). Overall, 58.3% of patients experienced total diagnostic delay, with a higher frequency among Thai patients (*p* = 0.036).

**Fig. 2:**
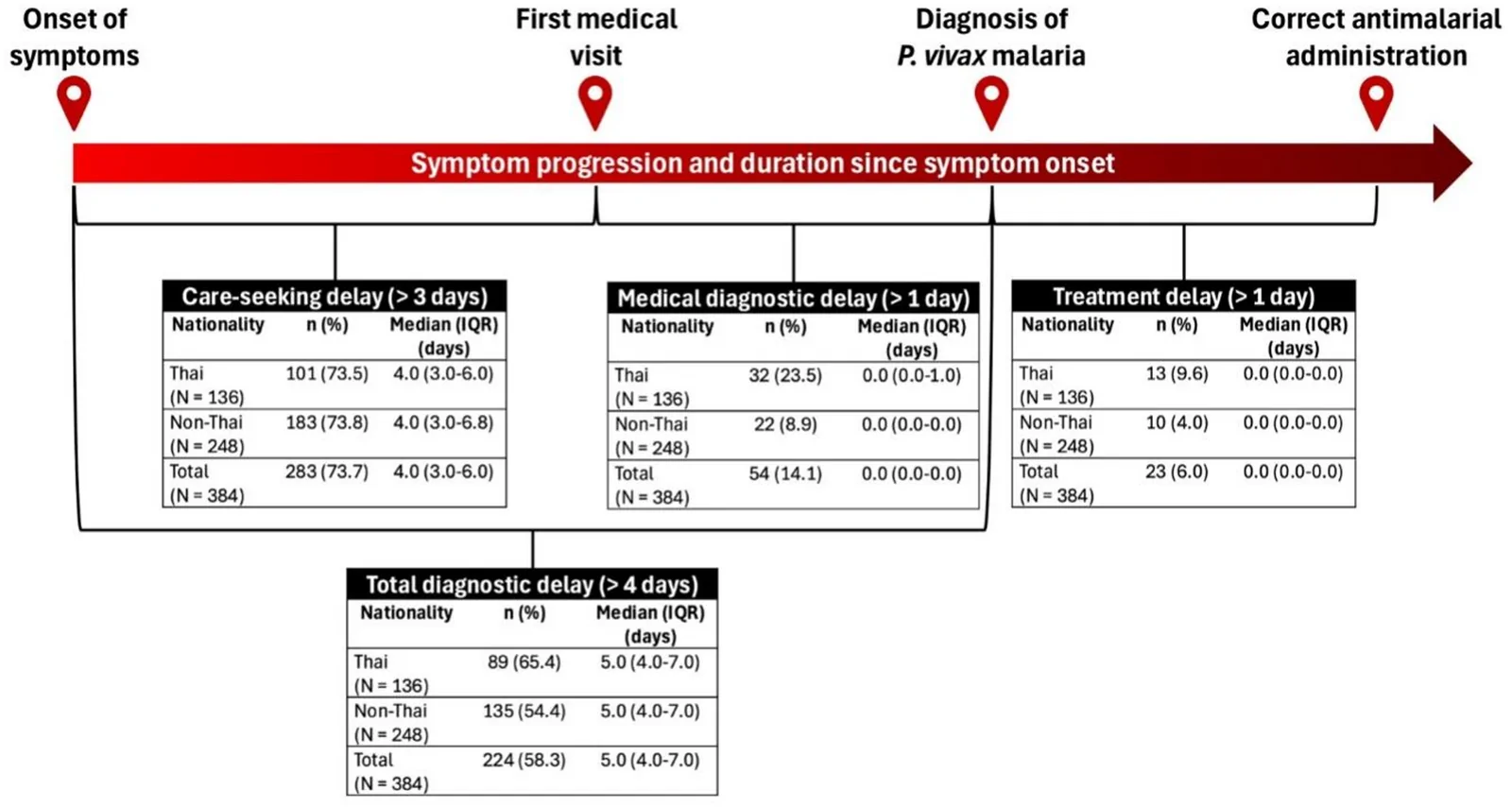
Frequency and duration of care-seeking, diagnostic, and treatment delays among Thai and non-Thai patients with *P. vivax* malaria

The median duration from diagnosis to antimalarial treatment initiation was 0.0 days (IQR 0.0–0.0) in both groups. Despite the identical median and IQR, the distributions differed between Thai and non-Thai patients (Mann-Whitney U test *p* = 0.033). Treatment delay occurred in 6.0% of patients and was more frequent among Thai patients (*p* = 0.029). Detailed distributions of all delay intervals are presented in Table 2; Fig. 2, and Supplementary Material S3.

The proportion of patients experiencing total diagnostic delay differed according to the first healthcare facility attended (*p* = 0.002). Higher proportion of delayed cases were observed among patients initially attending public hospitals (30.8% vs. 23.1%), primary care outpatient clinics (18.8% vs. 10.6%) and private hospitals (11.2% vs. 7.5%). In contrast, 58.8% of patients who first attended HTD did not experience total diagnostic delay. Non-Thai patients were more likely than Thais to attend HTD (51.6%) or primary care outpatient clinics (22.6%) (Table 2). Initial misdiagnosis was more frequent among Thai patients (*p* = 0.006) and was higher among patients with total diagnostic delay than those without delay (38.4% vs. 12.5%) (*p* < 0.001). Moreover, initial misdiagnosis was found predominantly in public hospitals (49.0%), followed by primary care outpatient clinics (34.7%) and private hospitals (15.3%). Detailed characteristics of first healthcare contact and initial diagnosis are presented in Table 1.

### Laboratory profiles on admission and clinical outcomes

Schizonts on blood film were more frequently observed among patients with delayed diagnosis than among those without delay (*p* = 0.048). Anemia (*p* < 0.001) and transaminitis (*p* < 0.001) were also common in the delayed diagnosis group.

In contrast, the frequencies of severe malaria on admission (*p* = 0.073), ICU admission (*p* = 0.074), oxygen supplementation (*p* = 0.967) or inotropic support (*p* = 0.807) did not differ significantly between groups. Laboratory findings and clinical trajectories are presented in Table 3.

### Factors associated with total delayed diagnosis of P. vivax malaria

Prior to multivariable analysis, multicollinearity was assessed; all variables had VIF < 3, indicating no evidence of problematic collinearity (Supplementary material S4). The multivariable model was adjusted for age, nationality, occupation, prior malaria infection, country of malaria acquisition, travel purpose, initial body temperature, headache, first healthcare facility, and initial diagnosis (Table 4; Supplementary material S5). Four factors were independently associated with total diagnostic delay: age 45–54 years (adjusted odds ratio [AOR] 2.79; 95% CI 1.12–6.94; *p* = 0.027), employment as employee/office worker/business owner (AOR 4.93; 95% CI 1.19–20.50; *p* = 0.028), absence of prior malaria infection (AOR 2.36; 95% CI 1.26–4.42; *p* = 0.008), and misdiagnosis at first healthcare facility (AOR 2.99; 95% CI 1.52–5.89; *p* = 0.002).

In a sensitivity analysis treating age as a continuous variable, each one-year increase in age was associated with higher odds of delayed diagnosis in univariable logistic regression (OR = 1.02, 95% CI: 1.01–1.04, *p* = 0.007), suggesting a consistent association between increasing age and delayed diagnosis.

A secondary analysis using a 6-day cut-off is shown in Supplementary material S6. Under the 6-day cut-off, 114 patients (29.7%) were classified as having delayed diagnosis, compared with 224 patients (58.3%) using the 4-day cut-off. Two factors were independently associated with total diagnostic delay at 6 days: malaria acquisition from Myanmar (AOR 2.48, 95% CI 1.26–4.87, *p* = 0.009) and misdiagnosis at first healthcare facility (AOR 2.25; 95% CI 1.20–4.20; *p* = 0.012). Other factors that were significant in the primary analysis using the 4-day cut-off, including age 45–54 years, occupation, and absence of prior malaria infection, were not statistically significant at the 6-day cut-off.

## Discussion

This study examined the epidemiology, outcomes, and associated factors of total diagnostic delay in *P. vivax* malaria in an urban Thai setting. As Thailand progresses towards malaria elimination, the number of imported *P. vivax* cases increased from 586 cases in 2021 to 7,246 in 2023 before declining to 4,516 in 2025 [13]. In urban areas, particularly Bangkok, most *P. vivax* cases were imported, either internationally from neighboring countries or domestically from provinces bordering Myanmar and Cambodia [13]. 

The diagnostic challenges observed in Thailand reflect many countries approaching malaria elimination. Under the WHO E-2025 initiative, 25 countries, including Thailand, Bhutan, Timor-Leste, Nepal, and Malaysia, were targeted for malaria elimination by 2025 [1]. Despite achieving zero indigenous transmission in some settings, such as Bhutan and Timor-Leste, timely recognition of imported malaria remains a key challenge in sustaining malaria-free status [1, 22]. In Kuala Lumpur, for example, imported *P. vivax* accounted for a substantial proportion of reported cases, underscoring the shared challenge of managing imported malaria in urbanized, near-elimination settings [23]. 

In this cohort, almost 60% of *P. vivax* patients experienced total diagnostic delay, a proportion higher than that reported in some previous studies [20, 24], but comparable to findings from France, Sri Lanka, and China where malaria cases were predominantly imported [11, 25–27]. Care-seeking delay was the primary contributor, accounting for 73% of cases, while medical diagnostic delay accounted for 14.1%. The median time from symptom onset to first medical visit was four days, exceeding the three-day care-seeking threshold and suggesting limited patient awareness of *P. vivax* malaria. Similar findings have been reported, with care-seeking delay accounting for most diagnostic delays [28]. Furthermore, misdiagnosis at the initial healthcare encounter is common in imported malaria (46–57%) and substantially contributes to prolonged diagnostic delays [16, 17]. 

Four factors were independently associated with total diagnostic delay in multivariable analysis: age 45–54 years, employment as an office worker or business owner, absence of prior malaria history, and initial misdiagnosis. These findings underscore the contribution of both patient and healthcare-system-level barriers. Urban workers and malaria-naïve individuals may have low perceived malaria risk and limited symptom awareness, resulting in delayed healthcare seeking [20, 28, 29]. In particular, office workers and business owners, whose predominantly urban lifestyles are associated with infrequent malaria exposure, may be less likely to suspect malaria after travel to endemic areas [20, 28]. Likewise, individuals without previous malaria experience may lack the illness familiarity that facilitates early symptom recognition and prompt healthcare seeking [30, 31]. 

At the healthcare system level, nearly one-quarter of patients were misdiagnosed at their initial healthcare encounter, reflecting insufficient clinician awareness of malaria in non-specialist settings [20, 28, 29, 32]. Notably, initial misdiagnosis in our cohort occurred predominantly in public hospitals, which were largely secondary or tertiary care centers, contrasting with previous reports in which misdiagnosis was more frequent in primary healthcare emergency settings [16]. As malaria transmission declines and becomes increasingly concentrated in limited geographical areas, clinicians in non-endemic settings may have fewer opportunities to encounter malaria cases. This reduced clinical exposure may contribute to decreased clinical suspicion and increase the risk of delayed diagnosis, particularly among patients with relevant travel histories [33–35]. Thus, routine assessment of travel and exposure history at triage remains a critical diagnostic step, even in metropolitan settings.

Although Thai patients experienced higher rates of total diagnostic delay than non-Thai patients on crude analysis (*p* = 0.036), this difference was not significant after adjustment for other covariates. The crude difference likely reflects the substantially different demographic and exposure profiles of the two groups rather than nationality per se. For instance, patients from neighboring countries in our cohort more frequently sought care directly at HTD, a specialized referral center with rapid diagnostic capacity, whereas Thai patients more often visited public or private hospitals first, likely influenced by insurance scheme differences. Additionally, patients from neighboring countries in this study may benefit from prior malaria exposure that enhances symptom recognition and prompts earlier care-seeking, and clinicians may maintain a higher index of suspicion for malaria in this group [34, 35]. Similar patterns have been reported in Canada and Spain, where presentation to specialized referral centers was associated with more accurate and timely diagnosis [16, 36]. A stratified analysis by nationality was not performed given the relatively small Thai subgroup (*n* = 136); larger future studies are warranted to characterize nationality-specific predictors of diagnostic delay.

Age 45–54 years was independently associated with total diagnostic delay, consistent with findings from China [20]. By contrast, age ≥ 55 years did not reach significance in the multivariable model, likely reflecting the small number of patients in this stratum (*n* = 25) and consequent wide confidence intervals. Previous studies have suggested that older adults, particularly those from endemic areas, may underestimate malaria risk, prefer watchful waiting, or attempt self-medication with antipyretics prior to seeking care [20, 37]. 

Different studies have used varying cut-offs (e.g. 3 or 4 days) and timepoints (e.g. from symptom onset to first healthcare consultation, hospital admission, or malaria diagnosis) to classify delay, showing that estimates and predictors can shift depending on the chosen threshold [16, 20, 25]. Our secondary analysis revealed that the 4-day and 6-day thresholds did not yield consistent findings in the multivariable model, with the exception of initial misdiagnosis at first healthcare facility visited. This finding further supports that initial misdiagnosis is a robust predictor of total diagnostic delay, particularly medical diagnostic delay, consistent with a previous study [20]. Clinically, this finding may be expected because failure to initially recognize malaria may lead to continued evaluation for alternative causes of fever, thereby prolonging the time to correct diagnosis. In contrast, the effects of other factors, such as age, occupation, and previous malaria history, may become less apparent as the illness progresses and additional clinical evaluations are performed. The differences observed between the 4-day and 6-day cut-offs may also partly reflect methodological factors, including the smaller number of delayed cases identify using the 6-day definition (29.7% versus 58.3%, respectively) which may have reduced statistical power and limited the ability to detect significant associations.

For future studies, a 4-day threshold is recommended when the aim is early identification of at-risk patients and cross-study comparability, while a 6-day threshold may be more appropriate when the focus is on prolonged diagnostic failure or prediction of severe clinical outcomes. Earlier studies on imported malaria encompassing all species have reported that *P. vivax* is associated with longer delays in care-seeking and diagnosis than *P. falciparum* infection [20, 24, 38]. Although the selected threshold may vary across studies, future prospective studies are needed to derive and validate a disease-specific definition of diagnostic delay for *P. vivax*, particularly in non-endemic settings. Thresholds established for *P. falciparum* may not be directly applicable to *P. vivax*, given differences in disease progression and the risk of severe outcomes. A *P. vivax*-specific threshold, validated against clinically meaningful outcomes, would facilitate comparisons across studies and identify patients who may benefit from targeted interventions.

Despite the high frequency of delayed diagnosis at the 4-day cut-off, severe malaria on admission and intensive care requirements did not differ significantly between delayed and non-delayed groups. Nevertheless, severe malaria according to WHO criteria was more common among patients with delayed diagnosis (17.0% versus 10.0%; *p* = 0.073) [21], suggesting a potentially clinically relevant association but the study may have been underpowered to detect it. Previous studies demonstrated an association between delayed diagnosis and severe imported malaria; [16, 20] therefore, these findings should be interpreted with caution. The generally less severe clinical course of *P. vivax* and the capacity of a specialized referral center to mitigate adverse outcomes after admission may also have attenuated observable differences between groups [20]. On admission, delayed cases more frequently exhibited schizonts, anemia, and transaminitis, reflecting more advanced parasite maturation after a longer duration of untreated infection [39]. 

Several limitations should be acknowledged. The retrospective design resulted in some missing data from referring facilities, and important socioeconomic determinants of care-seeking behavior could not be assessed. Moreover, variables reported by patients, such as the date of symptom onset and prior healthcare-seeking history, may also be subject to recall bias. In addition, because the study relied on documented travel histories, the exact country of malaria acquisition could not be definitively confirmed. Therefore, the reported country of exposure should be interpreted as the most likely location based on the available epidemiological information. Furthermore, relapse, recrudescence, and reinfection could not be reliably distinguished because molecular genotyping data were unavailable. This limitation may have resulted in misclassification of the presumed site of malaria acquisition, particularly for *P. vivax* infections that can recur due to activation of dormant hypnozoite stages.

Microscopy-based diagnosis may also have missed very low-density *P. vivax* infections below the detection limit, although all included patients had microscopically confirmed infection. Finally, as a national referral center, HTD may attract disproportionately complex or delayed cases, resulting in referral bias and potentially overestimating the prevalence of diagnostic delay compared with broader population. Nevertheless, the referral setting of HTD enabled comprehensive characterization of patients’ diagnostic pathways before presentation, which was essential for evaluating factors associated with delayed diagnosis.

Despite these limitations, this study provides insights into diagnostic delay patterns of *P. vivax* malaria in a non-endemic metropolitan setting over a 15-year period, contributing evidence relevant to malaria elimination efforts in urbanized, near-elimination contexts. Clinicians should maintain high clinical suspicion for malaria in any febrile patient with recent travel to endemic regions, particularly working-age employees, office workers, and business owners without previous malaria experience. Routine travel history should remain standard practice even in metropolitan healthcare settings. Moreover, given that diagnostic delays were most pronounced among Thai travelers, targeted public health messaging delivered through digital and social media platforms may help raise awareness of malaria risk associated with travel. Future multicenter studies including primary care and private sector settings are needed to better characterize the full spectrum of diagnostic delay across diverse healthcare environments. Qualitative studies exploring health-seeking behaviors including barriers to healthcare access and factors contributing to initial misdiagnosis, across patient-related, diagnostic and health-system domains are warranted to inform targeted interventions.

## Conclusion

Delayed *P. vivax* diagnosis remains common in this urban referral setting in Bangkok, Thailand, with care-seeking delay contributing more substantially than medical diagnostic delay. Delayed diagnosis was associated with older adults, employment as an office worker or business owner, no prior history of malaria, and initial misdiagnosis at the first healthcare facility. These findings highlight the importance of considering malaria in febrile patients with relevant travel or epidemiological exposure, particularly in urban settings approaching malaria elimination, to facilitate timely diagnosis and treatment.

**Table 1:** Characteristics of patients of delayed and non-delayed diagnosis of *P. vivax* malaria cases

Characteristics	Total (384 cases) *N* (%)	Non-delayed diagnosis (160 cases) *n* (%)	Delayed diagnosis (224 cases) *n* (%)	*P*-value
*Sex*				0.962
Male	326 (84.9)	136 (85.0)	190 (84.8)	
Female	58 (15.1)	24 (15.0)	34 (15.2)	
*Age (years)*				0.047*
15–24	152 (39.6)	77 (48.1)	75 (33.5)	
25–34	109 (28.4)	42 (26.3)	67 (29.9)	
35–44	63 (16.4)	23 (14.4)	40 (17.9)	
45–54	35 (9.1)	11 (6.9)	24 (10.7)	
≥ 55	25 (6.5)	7 (4.4)	18 (8.0)	
Median (IQR)	27 (21–37)	25 (20–35)	29 (22–40)	
*Nationality*				0.036*
Thai	136 (35.4)	47 (29.4)	89 (39.7)	
Non-Thai^A^	248 (64.6)	113 (70.6)	135 (60.3)	
*Occupation*				0.009*
Laborer/agriculturist/farmer	258 (67.2)	120 (75.0)	138 (61.6)	
Employee/office worker/business owner	78 (20.3)	24 (15.0)	54 (24.1)	
Worker in forested areas^B^	19 (4.9)	3 (1.9)	16 (7.1)	
Unemployed	29 (7.6)	13 (8.1)	16 (7.1)	
*Having comorbidities*				0.430
No	350 (91.1)	148 (92.5)	202 (90.2)	
Yes^C^	34 (8.9)	12 (7.5)	22 (9.8)	
*Prior history of malaria infection*				< 0.001*
≥ 2 times	70 (18.2)	42 (26.3)	28 (12.5)	
1 time	119 (31.0)	56 (35.0)	63 (28.1)	
No prior history of malaria	195 (50.8)	62 (38.8)	133 (59.4)	
*Country of P. vivax malaria acquisition*				0.288^+^
Thailand	226 (58.9)	98 (61.3)	128 (57.1)	
Myanmar	71 (18.5)	23 (14.4)	48 (21.4)	
Lao	55 (14.3)	22 (13.8)	33 (14.7)	
Cambodia	27 (7.0)	15 (9.4)	12 (5.4)	
Others ^D^	5 (1.3)	2 (1.3)	3 (1.3)	
*Purpose of travel to/stay in malaria endemic areas*				0.002*
No travel history (living in endemic areas)	244 (63.5)	119 (74.4)	125 (55.8)	
Visiting friends and relatives	60 (15.6)	19 (11.9)	41 (18.3)	
Tourism	49 (12.8)	12 (7.5)	37 (16.5)	
Work	31 (8.1)	10 (6.3)	21 (9.4)	
*Body temperature (ºC)*				0.073
< 38.0	274 (71.4)	122 (76.3)	152 (67.9)	
≥ 38.0	110 (28.6)	28 (23.8)	72 (32.1)	
*Respiratory rate (times/min)*				0.290
≤ 20	254 (66.1)	101 (63.1)	153 (68.3)	
> 20	130 (33.9)	59 (36.9)	71 (31.7)	
*Blood pressure (mmHg)*				0.231
< 90/60	14 (3.6)	8 (5.0)	6 (2.7)	
≥ 90/60	370 (96.4)	152 (95.0)	218 (97.3)	
*Pulse rate (beats per minute)*				0.488
≤ 100	308 (80.2)	131 (81.9)	177 (79.0)	
> 100	76 (19.8)	29 (18.1)	47 (21.0)	
*Initial symptoms* ^E^				
Chill	334 (87.0)	141 (88.1)	193 (86.2)	0.573
Myalgia	218 (56.8)	92 (57.5)	126 (56.3)	0.807
Headache	208 (54.2)	96 (60.0)	112 (50.0)	0.053
Loss of appetite	158 (41.1)	67 (41.9)	91 (40.6)	0.806
Weakness/fatigue	128 (33.3)	52 (32.5)	76 (33.9)	0.770
Nausea	124 (32.3)	56 (35.0)	68 (30.4)	0.337
Vomiting	75 (19.5)	29 (18.1)	46 (20.5)	0.557
Hepatosplenomegaly	68 (17.7)	27 (16.9)	41 (18.3)	0.718
Dizziness	64 (16.7)	29 (18.1)	35 (15.6)	0.517
Jaundice	54 (14.1)	23 (14.4)	31 (13.8)	0.882
Abdominal pain	46 (12.0)	21 (13.1)	25 (11.2)	0.559
Diarrhea	22 (5.7)	8 (5.0)	14 (6.3)	0.603
Pallor	15 (3.9)	4 (2.5)	11 (4.9)	0.229
Sweating	10 (2.6)	3 (1.9)	7 (3.1)	0.531^+^
Shortness of breath	10 (2.6)	3 (1.9)	7 (3.1)	0.531^+^
Oliguria/anuria	9 (2.3)	5 (3.1)	4 (1.8)	0.499^+^
Bleeding	6 (1.6)	3 (1.9)	3 (1.3)	1.000^+^
Impaired consciousness	5 (1.3)	3 (1.9)	2 (0.9)	0.653^+^
*Facility of first medical visit*				0.002*
Hospital for Tropical Diseases	182 (47.4)	94 (58.8)	88 (39.3)	
Public hospital	106 (27.6)	37 (23.1)	69 (30.8)	
Primary care outpatient clinics	59 (15.4)	17 (10.6)	42 (18.8)	
Private hospital	37 (9.6)	12 (7.5)	25 (11.2)	
*Diagnosis of P. vivax at first medical visit*				< 0.001*
Correct diagnosis	286 (74.5)	140 (87.5)	146 (65.2)	
Misdiagnosis	98 (25.5)	20 (12.5)	78 (34.8)	

*Statistical significant at 95% CI

^+^Using Fisher’s exact test for calculation. Other variables without markings at *p*-values used Pearson’s chi-square for calculation

^A^Includes Burmese, Laotian, and Cambodian

^B^Includes forest workers, patrol policeman and military personnels, pilgrim monks

^C^Includes hypertension, diabetes mellitus, cardiovascular diseases, non-malignancy hematologic diseases, hyperlipidemia, lung diseases, and liver diseases

^D^Includes Tanzania, Somalia, Pakistan, and Indonesia

^E^Most cases had more than one symptom

.

**Table 2:** Comparison of care-seeking behaviors and diagnosis and treatment delays in *P. vivax* cases between Thai and non-Thai patients

Variables	Total (384 cases) *N* (%)	Thai patients (136 cases) *n* (%)	Non-Thai patients (248 cases) *n* (%)	*P*-value
*Care-seeking delay (> 3 days)*				0.956
No	101 (26.3)	36 (26.5)	65 (26.2)	
Yes	283 (73.7)	101 (73.5)	183 (73.8)	
Median (IQR)	4.0 (3.0–6.0)	4.0 (3.0–6.0)	4.0 (3.0-6.8)	0.393^+^
*Medical diagnostic delay (> 1 day)*				< 0.001*
No	330 (85.9)	104 (76.5)	226 (91.1)	
Yes	54 (14.1)	32 (23.5)	42 (8.9)	
Median (IQR)	0.0 (0.0–0.0)	0.0 (0.0–1.0)	0.0 (0.0–0.0)	0.003*^+^
*Total diagnostic delay (> 4 days)*				0.036*
No	160 (41.7)	47 (34.6)	113 (45.6)	
Yes	224 (58.3)	89 (65.4)	135 (54.4)	
Median (IQR)	5.0 (4.0–7.0)	5.0 (4.0–7.0)	5.0 (4.0–7.0)	0.164^+^
*Treatment delay (> 1 day)*				0.029*
No	361 (94.0)	123 (90.4)	238 (96.0)	
Yes	23 (6.0)	13 (9.6)	10 (4.0)	
Median (IQR)	0.0 (0.0–0.0)	0.0 (0.0–0.0)	0.0 (0.0–0.0)	0.033*^+^
*Facility of first medical visit*				0.004*
Hospital for Tropical Diseases	182 (47.4)	54 (39.7)	128 (51.6)	
Public hospital	106 (27.6)	50 (36.8)	56 (22.6)	
Primary care outpatient clinics	59 (15.4)	15 (11.0)	44 (17.7)	
Private hospital	37 (9.6)	17 (12.5)	20 (8.1)	
*Diagnosis of P. vivax at first medical visit*				0.006*
Correct diagnosis	286 (74.5)	90 (66.2)	196 (79.0)	
Misdiagnosis	98 (25.5)	46 (33.8)	52 (21.0)	

*Significant at 95% CI

^+^Mann-Whitney U test

**Table 3:** Comparison of laboratory profiles on admission, severity of malaria on admission, and clinical course after admission at Hospital for Tropical Diseases between cases with delayed and non-delayed diagnosis

Laboratory profiles on admission, malaria severity on admission, and clinical course after admission at the Hospital for Tropical Diseases	Total (384 cases) *N* (%)	Non-delayed diagnosis (160 cases) *n* (%)	Delayed diagnosis (224 cases) *n* (%)	*P*-value
*Initial parasite density level (parasites/µL)*				0.300
< 1,000	59 (15.4)	25 (15.6)	34 (15.2)	
1,000–9,999	108 (28.1)	37 (23.1)	71 (31.7)	
10,000–19,999	98 (25.5)	43 (26.9)	55 (24.6)	
≥ 20,000	119 (31.0)	55 (34.4)	64 (28.6)	
*Presence of schizonts on admission*				0.048*
No	233 (60.7)	106 (66.3)	126 (56.3)	
Yes	152 (39.6)	54 (33.8)	98 (43.8)	
*White blood cells (cells/mm* ^*3*^ *)*				0.215
4,500 − 11,000	70 (18.2)	24 (15.0)	46 (20.5)	
< 4,500	302 (78.6)	129 (80.6)	173 (77.2)	
> 11,000	12 (3.1)	7 (4.4)	5 (2.2)	
*Anemia* ^A^				< 0.001*
No	175 (45.6)	93 (58.1)	82 (36.6)	
Yes	209 (54.4)	67 (41.9)	142 (63.4)	
*Platelet (platelets/mm* ^*3*^ *)*				0.360
150,000-400,000	52 (13.5)	26 (16.3)	26 (11.6)	
50,000-149,999	258 (67.2)	102 (63.7)	156 (69.6)	
< 50,000	74 (19.3)	32 (20.0)	42 (18.8)	
*Transaminitis (n = 361)* ^B^				< 0.001*
No	197 (54.6)	100 (65.8)	97 (46.4)	
Yes	164 (45.4)	52 (34.2)	112 (53.6)	
*Jaundice (mg/dL*,* n = 351)*				0.126
≤ 1.2	192 (54.7)	88 (59.5)	104 (51.2)	
> 1.2	159 (45.3)	60 (40.5)	99 (48.8)	
*Blood urea nitrogen (mg/dL*,* n = 360)*				0.291
6.0–20.0	313 (86.9)	132 (89.2)	181 (85.4)	
> 20.0	47 (13.1)	16 (10.8)	31 (14.6)	
*Creatinine (mg/dL*,* n = 361)*				0.885
0.5-1.0	226 (62.6)	92 (62.2)	134 (62.9)	
> 1.0	135 (37.4)	56 (37.8)	79 (37.1)	
*Serum sodium (mEq/L*,* n = 354)*				0.063^+^
136.0-145.0	253 (71.5)	116 (77.9)	137 (66.8)	
< 136.0	96 (27.1)	31 (20.8)	65 (31.7)	
> 145.0	5 (1.4)	2 (1.3)	3 (1.5)	
*Serum potassium (mEq/L*,* n = 354)*				0.055^+^
3.5–5.1	246 (69.5)	113 (75.8)	133 (64.9)	
< 3.5	106 (29.9)	35 (23.5)	71 (34.6)	
> 5.1	2 (0.6)	1 (0.7)	1 (0.5)	
*Serum chloride (mEq/L*,* n = 354)*				0.107
98–107	285 (80.5)	127 (85.2)	158 (77.1)	
< 98	52 (14.7)	15 (10.1)	37 (18.0)	
> 107	17 (4.8)	7 (4.7)	10 (4.9)	
*Serum bicarbonate* *(mEq/L*,* n = 354)*				0.478^+^
22–29	291 (82.2)	126 (84.6)	165 (80.5)	
< 22	57 (16.1)	20 (13.4)	37 (18.0)	
> 29	6 (1.7)	3 (2.0)	3 (1.5)	
*Severity of malaria on admission at Hospital for Tropical Diseases* ^C^				0.073
Non-severe malaria	330 (85.9)	144 (90.0)	186 (83.0)	
Severe malaria	54 (14.1)	16 (10.0)	38 (17.0)	
*Required admission to intensive care unit during admission at Hospital for Tropical Diseases*				0.074
No	356 (92.7)	153 (95.6)	203 (90.6)	
Yes	28 (7.3)	7 (4.4)	21 (9.4)	
*Required oxygen therapy during admission at Hospital for Tropical Diseases*				0.967
No	367 (95.6)	153 (95.6)	214 (95.5)	
Yes	17 (4.4)	7 (4.4)	10 (4.5)	
*Required inotropes during admission at Hospital for Tropical Diseases*				0.807
No	366 (95.3)	153 (95.6)	213 (95.1)	
Yes	18 (4.7)	7 (4.4)	11 (4.9)	
*Discharge status*				N/A
Improved	384 (100.0%)	160 (100.0%)	224 (100.0%)	
Died	0 (0.0%)	0 (0.0%)	0 (0.0%)	

*Significant at 95% CI

^+^Using Fisher’s exact test for calculation. Other variables without markings at *p*-values used Pearson’s chi-square for calculation

^A^Anemia was defined as hemoglobin (Hb) < 12.0 g/dL in females aged ≥ 15 years and < 13.0 g/dL in males aged ≥ 15 years [40]. This definition is distinct from the World Health Organization criterion for severe malarial anemia in adults (Hb ≤ 7.0 g/dL) [21]

^B^Defined as AST > 32 U/L or ALT > 33 U/L

^C^At least one of severe malaria criteria defined by World Health Organization’s guideline for malaria

**Table 4:** Univariable and multivariable analyses of factors associated with total diagnostic delay (> 4 days) among patients with *Plasmodium vivax* malaria

Variable	OR (95% CI)	*P*-value	Adjusted OR (95% CI)	*P*-value
*Age (years)*				
15–24	Reference		Reference	
25–34	1.64 (0.99–2.70)	0.053	1.63 (0.93–2.84)	0.088
35–44	1.79 (0.98–3.27)	0.060	1.63 (0.82–3.24)	0.166
45–54	2.24 (1.03–4.89)	0.043	2.79 (1.12–6.94)	0.027*
≥ 55	2.64 (1.04–6.69)	0.041	2.61 (0.81–8.42)	0.110
Age (per 1-year increase)	1.02 (1.01–1.04)	0.007		
*Nationality*				
Thai	Reference		Reference	
Non-Thai^A^	0.63 (0.41–0.97)	0.037	1.43 (0.64–3.18)	0.384
*Occupation*				
Laborer/agriculturist/farmer	Reference		Reference	
Employee/office worker/business owner	4.64 (1.32–16.30)	0.017	4.93 (1.19–20.50)	0.028*
Worker in forested areas ^B^	1.96 (1.14–3.36)	0.015	1.21 (0.57–2.56)	0.627
Unemployed	1.07 (0.50–2.32)	0.863	0.66 (0.24–1.82)	0.424
*Prior history of malaria infection*				
≥ 2 times	Reference		Reference	
1 time	1.69 (0.93–3.07)	0.087	1.71 (0.89–3.31)	0.110
No prior history of malaria	3.22 (1.83–5.66)	< 0.001	2.36 (1.26–4.42)	0.008*
*Country of P. vivax malaria acquisition*				
Thailand	Reference		Reference	
Myanmar	1.60 (0.91–2.80)	0.102	1.59 (0.81–3.15)	0.180
Lao	1.15 (0.63–2.09)	0.651	1.44 (0.69–3.03)	0.334
Cambodia	0.61 (0.27–1.37)	0.232	0.56 (0.21–1.52)	0.256
Others ^C^	1.15 (0.19–7.01)	0.881	0.73 (0.08–6.70)	0.783
*Purpose of travel to/stay in malaria endemic areas*				
No travel history (living in endemic areas)	Reference		Reference	
Visiting friends and relatives	2.05 (1.13–3.74)	0.019	1.30 (0.65–2.59)	0.456
Tourism	2.94 (1.46–5.90)	0.002	2.18 (0.83–5.69)	0.113
Work	2.00 (0.90–4.42)	0.087	1.49 (0.53–4.16)	0.446
*Body temperature (ºC)*				
< 38.0	Reference		Reference	
≥ 38.0	1.52 (0.96–2.41)	0.074	1.35 (0.79–2.29)	0.269
*Headache*				
No	Reference		Reference	
Yes	0.67 (0.44–1.01)	0.053	0.90 (0.55–1.48)	0.675
*Facility of first medical visit*				
Hospital for Tropical Diseases	Reference		Reference	
Public hospital	2.23 (1.05–4.70)	0.036	0.81 (0.33–1.99)	0.641
Primary care outpatient clinics	1.99 (1.22–3.27)	0.006	1.12 (0.59–2.15)	0.728
Private hospital	2.64 (1.40–4.98)	0.003	1.51 (0.68–3.36)	0.317
*Diagnosis of P. vivax at first medical visit*				
Correct diagnosis	Reference		Reference	
Misdiagnosis	3.74 (2.17–6.44)	< 0.001	2.99 (1.52–5.89)	0.002*

*Statistical significant at 95% CI

^A^Includes Burmese, Laotian, and Cambodian

^B^Includes forest workers, patrol policeman and military personnels, pilgrim monks

^C^Includes Tanzania, Somalia, Pakistan, and Indonesia

## Supplementary Information

Below is the link to the electronic supplementary material.

Supplementary Material 1

## Data Availability

No datasets were generated or analysed during the current study.
