# Supplementary material for: Delayed diagnosis of Plasmodium vivax malaria and associated factors among Thai patients and patients from neighboring countries at the Hospital for Tropical Diseases, Bangkok, Thailand
Source: Infection. 2026 Aug 17;54(5):2821–34. doi: 10.1007/s15010-026-02930-8 (PMC13727800; doi:10.1007/s15010-026-02930-8)
Supplement: Supplementary file 1 [file 15010_2026_2930_MOESM1_ESM.docx]

**S1.** ROC analysis showing optimal cut-off point for diagnostic delay

**S2.** Coordinates of the ROC curve

| Test Result Variable(s): Duration from symptoms to diagnosis of malaria (days) | | | |
| --- | --- | --- | --- |
| **Positive if greater than or equal to** | **Sensitivity** | **1 - Specificity** | **Youden's Index** |
| .0000 | 1.000 | 1.000 | .000 |
| 1.5000 | .981 | .982 | .000 |
| 2.5000 | .981 | .939 | .042 |
| 3.5000 | .926 | .797 | .129 |
| 4.5000 | .704 | .564 | .140 |
| 5.5000 | .593 | .397 | .196 |
| 6.5000 | .463 | .270 | .193 |
| 7.5000 | .278 | .212 | .066 |
| 8.5000 | .148 | .148 | .000 |
| 9.5000 | .093 | .127 | -.035 |
| 10.5000 | .056 | .115 | -.060 |
| 11.5000 | .056 | .097 | -.041 |
| 13.0000 | .056 | .094 | -.038 |
| 14.5000 | .056 | .073 | -.017 |
| 15.5000 | .019 | .048 | -.030 |
| 16.5000 | .019 | .042 | -.024 |
| 17.5000 | .019 | .039 | -.021 |
| 19.0000 | .019 | .033 | -.015 |
| 22.0000 | .019 | .024 | -.006 |
| 24.5000 | .019 | .021 | -.003 |
| 27.0000 | .000 | .021 | -.021 |
| 29.5000 | .000 | .018 | -.018 |
| 33.5000 | .000 | .006 | -.006 |
| 50.5000 | .000 | .003 | -.003 |
| 65.0000 | .000 | .000 | .000 |

**S3.** Distribution of care-seeking delay, medical diagnostic delay, total diagnostic delay, and treatment delay among Thai and non-Thai patients

| **Delay** | **Distribution and mean ranks** | **Mann-Whitney U, *p*-value** |
| --- | --- | --- |
| Care-seeking delay |  | Mann-Whitney U = 17,742.50, *p* = 0.393 |
| Medical diagnostic delay |  | Mann-Whitney U = 14,646.50, *p* = 0.003 |
| Total diagnostic delay |  | Mann-Whitney U = 15,431.00, *p* = 0.164 |
| Treatment delay |  | Mann-Whitney U = 15,952.00, *p* = 0.033 |

**S4.** Multicollinearity test results

| **Model** | **Collinearity Statistics** | |
| --- | --- | --- |
|  | **Tolerance** | **VIF** |
| Age | .742 | 1.348 |
| Prior history of malaria infection | .914 | 1.094 |
| Nationality | .402 | 2.486 |
| Body temperature | .903 | 1.107 |
| Purpose of travel | .649 | 1.541 |
| First medical visit | .630 | 1.587 |
| Country of malaria acquisition | .727 | 1.376 |
| Headache | .823 | 1.215 |
| Occupation | .603 | 1.658 |
| Initial diagnosis | .654 | 1.529 |

**S5.** Univariable and multivariable logistic regression of factors associated with *P. vivax* total diagnostic delay (> 4 days) in Hospital for Tropical Diseases, Bangkok, Thailand.

| **Variable** | **Total diagnostic delay (> 4 days)** | | **OR (95% CI)** | ***P*-value** | **Adjusted OR (95% CI)** | ***P*-value** |
| --- | --- | --- | --- | --- | --- | --- |
|  | **No**  **n (%)** | **Yes**  **n (%)** |  |  |  |  |
| **Gender** | | |  |  |  |  |
| Male | 136 (85.0) | 190 (84.8) | Reference |  |  |  |
| Female | 24 (15.0) | 34 (15.2) | 1.01 (0.58-1.79) | 0.962 |  |  |
| **Age (years)** | | |  |  |  |  |
| 15-24 | 77 (48.1) | 75 (33.5) | Reference |  | Reference |  |
| 25-34 | 42 (26.3) | 67 (29.9) | 1.64 (0.99-2.70) | 0.053 | 1.63 (0.93-2.84) | 0.088 |
| 35-44 | 23 (14.4) | 40 (17.9) | 1.79 (0.98-3.27) | 0.060 | 1.63 (0.82-3.24) | 0.166 |
| 45-54 | 11 (6.9) | 24 (10.7) | 2.24 (1.03-4.89) | 0.043 | 2.79 (1.12-6.94) | 0.027* |
| ≥ 55 | 7 (4.4) | 18 (8.0) | 2.64 (1.04-6.69) | 0.041 | 2.61 (0.81-8.42) | 0.110 |
| **Nationality** | | |  |  |  |  |
| Thai | 47 (29.4) | 89 (39.7) | Reference |  | Reference |  |
| Migrants ^A^ | 113 (70.6) | 135 (60.3) | 0.63 (0.41-0.97) | 0.037 | 1.43 (0.64-3.18) | 0.384 |
| **Occupation** | | |  |  |  |  |
| Laborer/agriculturist/farmer | 120 (75.0) | 138 (61.6) | Reference |  | Reference |  |
| Employee/office worker/business owner | 24 (15.0) | 54 (24.1) | 4.64 (1.32-16.30) | 0.017 | 4.93 (1.19-20.50) | 0.028* |
| Worker in forested areas ^B^ | 3 (1.9) | 16 (7.1) | 1.96 (1.14-3.36) | 0.015 | 1.21 (0.57-2.56) | 0.627 |
| Unemployed | 13 (8.1) | 16 (7.1) | 1.07 (0.50-2.32) | 0.863 | 0.66 (0.24-1.82) | 0.424 |
| **Having comorbidities** | | |  |  |  |  |
| No | 148 (92.5) | 202 (90.2) | Reference |  |  |  |
| Yes ^C^ | 12 (7.5) | 22 (9.8) | 1.34 (0.64-2.80) | 0.431 |  |  |
| **Prior history of malaria infection** | | |  |  |  |  |
| ≥ 2 times | 42 (26.3) | 28 (12.5) | Reference |  | Reference |  |
| 1 time | 56 (35.0) | 63 (28.1) | 1.69 (0.93-3.07) | 0.087 | 1.71 (0.89-3.31) | 0.110 |
| No prior history of malaria | 45 (28.1) | 101 (45.1) | 3.22 (1.83-5.66) | < 0.001 | 2.36 (1.26-4.42) | 0.008* |
| **Country of *P. vivax* malaria acquisition** | | |  |  |  |  |
| Thailand | 98 (61.3) | 128 (57.1) | Reference |  | Reference |  |
| Myanmar | 23 (14.4) | 48 (21.4) | 1.60 (0.91-2.80) | 0.102 | 1.59 (0.81-3.15) | 0.180 |
| Lao | 22 (13.8) | 33 (14.7) | 1.15 (0.63-2.09) | 0.651 | 1.44 (0.69-3.03) | 0.334 |
| Cambodia | 15 (9.4) | 12 (5.4) | 0.61 (0.27-1.37) | 0.232 | 0.56 (0.21-1.52) | 0.256 |
| Others ^D^ | 2 (1.3) | 3 (1.3) | 1.15 (0.19-7.01) | 0.881 | 0.73 (0.08-6.70) | 0.783 |
| **Purpose of travel to/stay in malaria endemic areas** | | |  |  |  |  |
| No travel history (living in endemic areas) | 119 (74.4) | 125 (55.8) | Reference |  | Reference |  |
| Visiting friends and relatives | 19 (11.9) | 41 (18.3) | 2.05 (1.13-3.74) | 0.019 | 1.30 (0.65-2.59) | 0.456 |
| Tourism | 12 (7.5) | 37 (16.5) | 2.94 (1.46-5.90) | 0.002 | 2.18 (0.83-5.69) | 0.113 |
| Work | 10 (6.3) | 21 (9.4) | 2.00 (0.90-4.42) | 0.087 | 1.49 (0.53-4.16) | 0.446 |
| **Body temperature (ºC)** | | |  |  |  |  |
| < 38.0 | 122 (76.3) | 152 (67.9) | Reference |  | Reference |  |
| ≥ 38.0 | 28 (23.8) | 72 (32.1) | 1.52 (0.96-2.41) | 0.074 | 1.35 (0.79-2.29) | 0.269 |
| **Respiratory rate (times/min)** | | |  |  |  |  |
| ≤ 20 | 101 (63.1) | 153 (68.3) | Reference |  |  |  |
| > 20 | 59 (36.9) | 71 (31.7) | 0.79 (0.52-1.22) | 0.291 |  |  |
| **Blood pressure (mmHg)** | | |  |  |  |  |
| ≥ 90/60 | 152 (95.0) | 218 (97.3) | Reference |  |  |  |
| < 90/60 | 8 (5.0) | 6 (2.7) | 0.52 (0.18-1.54) | 0.239 |  |  |
| **Pulse rate (beats per minute)** | | |  |  |  |  |
| ≤ 100 | 131 (81.9) | 177 (79.0) | Reference |  |  |  |
| > 100 | 29 (18.1) | 47 (21.0) | 1.20 (0.72-2.01) | 0.489 |  |  |
| **Initial symptoms ^E^** | | |  |  |  |  |
| Chill |  |  |  |  |  |  |
| No | 19 (11.9) | 31 (13.8) | Reference |  |  |  |
| Yes | 141 (88.1) | 193 (86.2) | 0.84 (0.46-1.55) | 0.573 |  |  |
| Myalgia |  |  |  |  |  |  |
| No | 68 (42.5) | 98 (43.8) | Reference |  |  |  |
| Yes | 92 (57.5) | 126 (56.3) | 0.95 (0.63-1.43) | 0.807 |  |  |
| Headache |  |  |  |  |  |  |
| No | 64 (40.0) | 112 (50.0) | Reference |  | Reference |  |
| Yes | 96 (60.0) | 112 (50.0) | 0.67 (0.44-1.01) | 0.053 | 0.90 (0.55-1.48) | 0.675 |
| Loss of appetite |  |  |  |  |  |  |
| No | 93 (58.1) | 133 (59.4) | Reference |  |  |  |
| Yes | 67 (41.9) | 91 (40.6) | 0.95 (0.63-1.43) | 0.806 |  |  |
| Weakness/fatigue |  |  |  |  |  |  |
| No | 108 (67.5) | 148 (66.1) | Reference |  |  |  |
| Yes | 52 (32.5) | 76 (33.9) | 1.07 (0.69-1.64) | 0.770 |  |  |
| Nausea |  |  |  |  |  |  |
| No | 104 (65.0) | 156 (69.6) | Reference |  |  |  |
| Yes | 56 (35.0) | 68 (30.4) | 0.81 (0.53-1.25) | 0.338 |  |  |
| Vomiting |  |  |  |  |  |  |
| No | 131 (81.9) | 178 (79.5) | Reference |  |  |  |
| Yes | 29 (18.1) | 46 (20.5) | 1.17 (0.70-1.96) | 0.557 |  |  |
| Hepatosplenomegaly |  |  |  |  |  |  |
| No | 133 (83.1) | 183 (81.7) | Reference |  |  |  |
| Yes | 27 (16.9) | 41 (18.3) | 1.10 (0.65-1.88) | 0.718 |  |  |
| Dizziness |  |  |  |  |  |  |
| No | 131 (81.9) | 189 (84.4) | Reference |  |  |  |
| Yes | 29 (18.1) | 35 (15.6) | 0.84 (0.49-1.44) | 0.517 |  |  |
| Jaundice |  |  |  |  |  |  |
| No | 137 (85.6) | 193 (86.2) | Reference |  |  |  |
| Yes | 23 (14.4) | 31 (13.8) | 0.96 (0.54-1.71) | 0.882 |  |  |
| Abdominal pain |  |  |  |  |  |  |
| No | 139 (86.9) | 199 (88.8) | Reference |  |  |  |
| Yes | 21 (13.1) | 25 (11.2) | 0.83 (0.45-1.55) | 0.559 |  |  |
| Diarrhea |  |  |  |  |  |  |
| No | 152 (95.0) | 210 (93.8) | Reference |  |  |  |
| Yes | 8 (5.0) | 14 (6.3) | 1.27 (0.52-3.10) | 0.604 |  |  |
| Pallor |  |  |  |  |  |  |
| No | 156 (97.5) | 213 (95.1) | Reference |  |  |  |
| Yes | 4 (2.5) | 11 (4.9) | 2.01 (0.63-6.44) | 0.238 |  |  |
| Sweating |  |  |  |  |  |  |
| No | 157 (98.1) | 217 (96.9) | Reference |  |  |  |
| Yes | 3 (1.9) | 7 (3.1) | 1.69 (0.43-6.63) | 0.453 |  |  |
| Shortness of breath |  |  |  |  |  |  |
| No | 157 (98.1) | 217 (96.9) | Reference |  |  |  |
| Yes | 3 (1.9) | 7 (3.1) | 1.69 (0.43-6.63) | 0.453 |  |  |
| Oliguria/anuria |  |  |  |  |  |  |
| No | 155 (96.9) | 220 (98.2) | Reference |  |  |  |
| Yes | 5 (3.1) | 4 (1.8) | 0.56 (0.15-2.13) | 0.398 |  |  |
| Bleeding |  |  |  |  |  |  |
| No | 157 (98.1) | 221 (98.7) | Reference |  |  |  |
| Yes | 3 (1.9) | 3 (1.3) | 0.71 (0.14-3.57) | 0.678 |  |  |
| Impaired consciousness |  |  |  |  |  |  |
| No | 157 (98.1) | 222 (99.1) | Reference |  |  |  |
| Yes | 3 (1.9) | 2 (0.9) | 0.47 (0.08-2.86) | 0.413 |  |  |
| **Facility of first medical visit** | | |  |  |  |  |
| Hospital for Tropical Diseases | 59 (58.8) | 88 (39.3) | Reference |  | Reference |  |
| Public hospital | 69 (30.8) | 106 (27.6) | 2.23 (1.05-4.70) | 0.036 | 0.81 (0.33-1.99) | 0.641 |
| Primary care outpatient clinics | 17 (10.6) | 42 (18.8) | 1.99 (1.22-3.27) | 0.006 | 1.12 (0.59-2.15) | 0.728 |
| Private hospital | 12 (7.5) | 25 (11.2) | 2.64 (1.40-4.98) | 0.003 | 1.51 (0.68-3.36) | 0.317 |
| **Diagnosis of *P. vivax* at first medical visit** | | |  |  |  |  |
| Correct diagnosis | 140 (87.5) | 146 (65.2) | Reference |  | Reference |  |
| Misdiagnosis | 20 (12.5) | 78 (34.8) | 3.74 (2.17-6.44) | < 0.001 | 2.99 (1.52-5.89) | 0.002* |
| ^A^ Includes Myanmese, Laotian, and Cambodian  ^B^ Includes forest workers, patrol policeman and military personnels, pilgrim monks  ^C^ Includes hypertension, diabetes mellitus, cardiovascular diseases, non-malignancy hematologic diseases, hyperlipidemia, lung diseases, and liver diseases  ^D^ Includes Tanzania, Somalia, Pakistan, and Indonesia  ^E^ Most cases had more than one symptom  * Statistical significant at 95% CI | | | | | | |

**S6.** Secondary analysis of factors associated with *P. vivax* total diagnostic delay by using a 6-day cut-off (> 6 days)

| **Variable** | **Total diagnostic delay (> 6 days)** | | **OR (95% CI)** | ***P*-value** | **Adjusted OR (95% CI)** | ***P*-value** |
| --- | --- | --- | --- | --- | --- | --- |
|  | **No**  **n (%)** | **Yes**  **n (%)** |  |  |  |  |
| **Gender** | | |  |  |  |  |
| Male | 232 (85.9) | 94 (82.5) | Reference |  |  |  |
| Female | 38 (14.1) | 20 (17.5) | 1.30 (0.72-2.35) | 0.387 |  |  |
| **Age (years)** | | |  |  |  |  |
| 15-24 | 108 (40.0) | 44 (38.6) | Reference |  |  |  |
| 25-34 | 75 (27.8) | 34 (29.8) | 1.11 (0.65-1.9) | 0.696 |  |  |
| 35-44 | 44 (16.3) | 19 (16.7) | 1.06 (0.56-2.01) | 0.859 |  |  |
| 45-54 | 24 (8.9) | 11 (9.6) | 1.13 (0.51-2.49) | 0.772 |  |  |
| ≥ 55 | 19 (7.0) | 6 (5.3) | 0.78 (0.29-2.07) | 0.611 |  |  |
| **Nationality** | | |  |  |  |  |
| Thai | 95 (35.2) | 41 (36.0) | Reference |  |  |  |
| Migrants ^A^ | 175 (64.8) | 73 (64.0) | 0.97 (0.61-1.53) | 0.884 |  |  |
| **Occupation** | | |  |  |  |  |
| Laborer/agriculturist/farmer | 188 (69.6) | 70 (61.4) | Reference |  | Reference |  |
| Employee/office worker/business owner | 51 (18.9) | 27 (23.7) | 2.42 (0.94-6.20) | 0.066 | 2.44 (0.88-6.73) | 0.086 |
| Worker in forested areas ^B^ | 10 (3.7) | 9 (7.9) | 1.42 (0.83-2.44) | 0.202 | 1.24 (0.64-2.38) | 0.526 |
| Unemployed | 21 (7.8) | 8 (7.0) | 1.023 (0.43-2.42) | 0.958 | 0.86 (0.33-2.26) | 0.766 |
| **Having comorbidities** | | |  |  |  |  |
| No | 249 (92.2) | 101 (88.6) | Reference |  |  |  |
| Yes ^C^ | 21 (7.8) | 13 (11.4) | 1.53 (0.74-3.17) | 0.256 |  |  |
| **Prior history of malaria infection** | | |  |  |  |  |
| ≥ 2 times | 53 (19.6) | 17 (14.9) | Reference |  | Reference |  |
| 1 time | 90 (33.3) | 29 (25.4) | 1.00 (0.51-2.00) | 0.990 | 0.96 (0.45-2.03) | 0.904 |
| No prior history of malaria | 127 (47.0) | 68 (59.6) | 1.67 (0.90-3.11) | 0.106 | 1.24 (0.62-2.50) | 0.545 |
| **Country of *P. vivax* malaria acquisition** | | |  |  |  |  |
| Thailand | 169 (62.6) | 57 (50.0) | Reference |  | Reference |  |
| Myanmar | 45 (16.7) | 26 (22.8) | 1.71 (0.97-3.03) | 0.064 | 1.50 (0.77-2.89) | 0.231 |
| Lao | 31 (11.5) | 24 (21.1) | 2.30 (1.25-4.23) | 0.008 | 2.48 (1.26-4.87) | 0.009* |
| Cambodia | 21 (7.8) | 6 (5.3) | 0.85 (0.33-2.20) | 0.734 | 0.71 (0.25-2.05) | 0.526 |
| Others ^D^ | 4 (1.5) | 1 (0.9) | 0.74 (0.08-6.77) | 0.791 | 0.53 (0.50-5.63) | 0.600 |
| **Purpose of travel to/stay in malaria endemic areas** | | |  |  |  |  |
| No travel history (living in endemic areas) | 177 (65.6) | 67 (58.8) | Reference |  |  |  |
| Visiting friends and relatives | 39 (14.4) | 21 (18.4) | 1.42 (0.78-2.59) | 0.250 |  |  |
| Tourism | 33 (12.2) | 16 (14.0) | 1.28 (0.66-2.48) | 0.462 |  |  |
| Work | 21 (7.8) | 10 (8.8) | 1.26 (0.56-2.81) | 0.576 |  |  |
| **Body temperature (ºC)** | | |  |  |  |  |
| < 38.0 | 195 (72.2) | 79 (69.3) | Reference |  |  |  |
| ≥ 38.0 | 75 (27.8) | 35 (30.7) | 1.15 (0.71-1.86) | 0.563 |  |  |
| **Respiratory rate (times/min)** | | |  |  |  |  |
| ≤ 20 | 174 (64.4) | 80 (70.2) | Reference |  |  |  |
| > 20 | 96 (35.6) | 34 (29.8) | 0.77 (0.48-1.24) | 0.279 |  |  |
| **Blood pressure (mmHg)** | | |  |  |  |  |
| ≥ 90/60 | 259 (95.9) | 111 (97.4) | Reference |  |  |  |
| < 90/60 | 11 (4.1) | 3 (2.6) | 0.64 (0.17-2.33) | 0.494 |  |  |
| **Pulse rate (beats per minute)** | | |  |  |  |  |
| ≤ 100 | 222 (82.2) | 86 (75.4) | Reference |  | Reference |  |
| > 100 | 48 (17.8) | 28 (24.6) | 1.51 (0.89-2.55) | 0.129 | 1.27 (0.70-2.29) | 0.433 |
| **Initial symptoms ^E^** | | |  |  |  |  |
| Chill |  |  |  |  |  |  |
| No | 35 (13.0) | 15 (13.2) | Reference |  |  |  |
| Yes | 235 (87.0) | 99 (86.8) | 0.98 (0.51-1.88) | 0.959 |  |  |
| Myalgia |  |  |  |  |  |  |
| No | 116 (43.0) | 50 (43.9) | Reference |  |  |  |
| Yes | 154 (57.0) | 64 (56.1) | 0.96 (0.62-1.50) | 0.871 |  |  |
| Headache |  |  |  |  |  |  |
| No | 114 (42.2) | 62 (54.4) | Reference |  | Reference |  |
| Yes | 156 (57.8) | 52 (45.6) | 0.61 (0.39-0.95) | 0.029 | 0.71 (0.42-1.19) | 0.191 |
| Loss of appetite |  |  |  |  |  |  |
| No | 156 (57.8) | 70 (61.4) | Reference |  |  |  |
| Yes | 114 (42.2) | 44 (38.6) | 0.86 (0.55-1.35) | 0.510 |  |  |
| Weakness/fatigue |  |  |  |  |  |  |
| No | 179 (66.3) | 77 (67.5) | Reference |  |  |  |
| Yes | 91 (33.7) | 37 (32.5) | 0.95 (0.59-1.51) | 0.813 |  |  |
| Nausea |  |  |  |  |  |  |
| No | 181 (67.0) | 79 (69.3) | Reference |  |  |  |
| Yes | 89 (33.0) | 35 (30.7) | 0.90 (0.56-1.44) | 0.665 |  |  |
| Vomiting |  |  |  |  |  |  |
| No | 218 (80.7) | 91 (79.8) | Reference |  |  |  |
| Yes | 52 (19.3) | 23 (20.2) | 1.06 (0.61-1.83) | 0.836 |  |  |
| Hepatosplenomegaly |  |  |  |  |  |  |
| No | 227 (84.1) | 89 (78.1)) | Reference |  | Reference |  |
| Yes | 43 (15.9) | 25 (21.9) | 1.48 (0.86-2.57) | 0.161 | 1.79 (0.96-3.33) | 0.067 |
| Dizziness |  |  |  |  |  |  |
| No | 217 (80.4) | 103 (90.4) | Reference |  | Reference |  |
| Yes | 53 (19.6) | 11 (9.6) | 0.44 (0.22-0.88) | 0.019 | 0.67 (0.30-1.49) | 0.325 |
| Jaundice |  |  |  |  |  |  |
| No | 234 (86.7) | 96 (84.2) | Reference |  |  |  |
| Yes | 36 (13.3) | 18 (15.8) | 1.22 (0.66-2.25) | 0.527 |  |  |
| Abdominal pain |  |  |  |  |  |  |
| No | 235 (87.0) | 103 (90.4) | Reference |  |  |  |
| Yes | 35 (13.0) | 11 (9.6) | 0.72 (0.35-1.47) | 0.363 |  |  |
| Diarrhea |  |  |  |  |  |  |
| No | 253 (93.7) | 109 (95.6) | Reference |  |  |  |
| Yes | 17 (6.3) | 5 (4.4) | 0.68 (0.25-1.90) | 0.464 |  |  |
| Pallor |  |  |  |  |  |  |
| No | 262 (97.0) | 107 (93.9) | Reference |  | Reference |  |
| Yes | 8 (3.0) | 7 (6.1) | 2.14 (0.76-6.06) | 0.151 | 1.95 (0.63-6.05) | 0.25 |
| Sweating |  |  |  |  |  |  |
| No | 264 (97.8) | 110 (96.5) | Reference |  |  |  |
| Yes | 6 (2.2) | 4 (3.5) | 1.60 (0.44-5.78) | 0.473 |  |  |
| Shortness of breath |  |  |  |  |  |  |
| No | 266 (98.5) | 108 (94.7) | Reference |  | Reference |  |
| Yes | 4 (1.5) | 6 (5.3) | 3.69 (1.02-13.35) | 0.046 | 2.22 (0.54-9.15) | 0.27 |
| Oliguria/anuria |  |  |  |  |  |  |
| No | 263 (97.4) | 112 (98.2) | Reference |  |  |  |
| Yes | 7 (2.6) | 2 (1.8) | 0.67 (0.14-3.28) | 0.622 |  |  |
| Bleeding |  |  |  |  |  |  |
| No | 267 (98.9) | 111 (97.4) | Reference |  |  |  |
| Yes | 3 (1.1) | 3 (2.6) | 2.41 (0.48-12.10) | 0.287 |  |  |
| Impaired consciousness |  |  |  |  |  |  |
| No | 266 (98.5) | 113 (99.1) | Reference |  |  |  |
| Yes | 4 (1.5) | 1 (0.9) | 0.59 (0.07-5.32) | 0.637 |  |  |
| **Facility of first medical visit** | | |  |  |  |  |
| Hospital for Tropical Diseases | 146 (54.1) | 36 (31.6) | Reference |  | Reference |  |
| Public hospital | 67 (24.8) | 39 (34.2) | 1.95 (0.89-4.24) | 0.094 | 0.79 (0.31-1.99) | 0.619 |
| Primary care outpatient clinics | 32 (11.9) | 27 (23.7) | 2.36 (1.38-4.04) | 0.002 | 1.20 (0.61-2.35) | 0.604 |
| Private hospital | 25 (9.3) | 12 (10.5) | 3.43 (1.83-6.42) | < 0.001 | 1.66 (0.76-3.62) | 0.201 |
| **Diagnosis of *P. vivax* at first medical visit** | | |  |  |  |  |
| Correct diagnosis | 218 (80.7) | 68 (59.6) | Reference |  | Reference |  |
| Misdiagnosis | 52 (19.3) | 46 (40.4) | 2.84 (1.75-4.59) | < 0.001 | 2.25 (1.20-4.20) | 0.012* |
| ^A^ Includes Myanmese, Laotian, and Cambodian  ^B^ Includes forest workers, patrol policeman and military personnels, pilgrim monks  ^C^ Includes hypertension, diabetes mellitus, cardiovascular diseases, non-malignancy hematologic diseases, hyperlipidemia, lung diseases, and liver diseases  ^D^ Includes Tanzania, Somalia, Pakistan, and Indonesia  ^E^ Most cases had more than one symptom  * Statistical significant at 95% CI | | | | | | |
